# Sulfasalazine induces autophagy inhibiting neointimal hyperplasia following carotid artery injuries in mice

**DOI:** 10.3389/fbioe.2023.1199785

**Published:** 2023-05-23

**Authors:** Weichang Zhang, Cheng Yan, Yangyan Xiao, Yuxin Sun, Youjun Lin, Qinglong Li, Wenwu Cai

**Affiliations:** ^1^ Department of Vascular Surgery, Second Xiangya Hospital, Central South University, Changsha, China; ^2^ Department of General Surgery, Second Xiangya Hospital, Central South University, Changsha, China; ^3^ Department of Ophthalmology, Second Xiangya Hospital, Central South University, Changsha, China

**Keywords:** sulfasalazine, nanoparticles, neointimal hyperplasia, VSMCs, NF-kB, mTOR, carotid ligation, autophagy

## Abstract

**Background:** Neointimal hyperplasia (NH) is a crucial pathophysiological feature in vascular transplant and in-stent restenosis. Excessive proliferation and migration of vascular smooth muscle cells (VSMCs) play important roles in neointimal hyperplasia. This study aims to explore the potentialities and mechanism of sulfasalazine (SSZ) in the prevention of restenosis.

**Methods:** Sulfasalazine was encapsulated in nanoparticles made of poly (lactic-co-glycolic acid) (PLGA). *In vivo*, carotid ligation injury was induced in mice to induce Neointimal hyperplasia, with or without sulfasalazine containing nanoparticles (NP-SSZ) treatment. After 4 weeks, the arteries were collected for histology, immunofluorescence, Western blotting (WB) and qRT-PCR. *In vitro*, vascular smooth muscle cells were treated with TNF-α to induce cell proliferation and migration, followed by SSZ or vehicle treatment. WB was performed to further explore its mechanism.

**Results:** The ratio of intima to media thickness (I/M) was increased after ligation injury on day 28, while the ratio was significantly reduced in the NP-SSZ treatment group. The dual positive nuclei of Ki-67 and α-SMA were 47.83% ± 9.15%, whereas only 29.83% ± 5.98% in the NP-SSZ-treated group (*p* < 0.05). Both MMP-2 and MMP-9 were decreased in the NP-SSZ treatment group (*p* < 0.05, *p* < 0.05, respectively) compared to the control group. The levels of the targeted inflammatory genes (TNF-α, VCAM-1, ICAM-1, MCP-1) were lower in the NP-SSZ treatment group compared with the control group. *In vitro*, the proliferating cell nuclear antigen (PCNA) expression was significantly decreased in the SSZ treatment group. The cell viability of VSMCs was markedly increased in the TNF-α treatment group, whereas sulfasalazine treatment inhibited this effect. LC3 II and P62 protein expression were higher in the SSZ group than in the vehicle group both *in vitro* and *in vivo*. The phosphorylation of NF-kB (p-NF-kB) and the phosphorylation of mTOR (p-mTOR) were decreased in the TNF-α+ SSZ group, whereas the P62 and LC3 II expression levels were increased. However, the expression level of p-mTOR, P62, and LC3 II was reversed after co-treatment with the agonist of mTOR MHY1485, whereas the p-NF-kB expression level was unchanged.

**Conclusion:** sulfasalazine inhibited vascular smooth muscle cells proliferation and migration *in vitro* and Neointimal hyperplasia *in vivo* through NF-kB/mTOR-mediated autophagy.

## Introduction

Vascular restenosis is a persistent challenge in clinical practice for treating transplant organs or grafts and vascular angioplasty disease. Neointimal hyperplasia (NH), caused by inflammatory and proliferative responses of vascular smooth muscle cells (VSMCs) following vessel injury, is one of the major driving forces for restenosis (Owens et al., 2004). Although drug-eluting stents or balloons that deliver rapamycin and paclitaxel to inhibit neointimal hyperplasia are preferentially used to treat high-risk patients, over 80% of the intended drug dose may be lost in balloon transit or inflation (Heldman et al., 2001; Suzuki et al., 2001; Grube et al., 2003; Spaulding, 2022). Furthermore, these technologies are not effective for treating neointimal hyperplasia after traditional open surgical procedures. To address these limitations and enhance pharmacological treatment efficacy, polymeric nanoparticles (NPs) have been proposed as they can localize and sustain the drug concentration at the injured vessel (Iyer et al., 2019; Chen et al., 2021; Shah and Chandra, 2022).

Autophagy is a catabolic process that maintains intracellular homeostasis and regulates cell metabolism (Mizushima et al., 2011). Studies have shown that autophagy is involved in cardiovascular diseases, such as vascular aging (LaRocca et al., 2013), atherosclerosis (Martinet and De Meyer, 2009; He et al., 2013), and vascular restenosis (Grootaert et al., 2015). Autophagy is also associated with the proliferation and migration of VSMCs (Rensen et al., 2007). Sulfasalazine (SSZ) is an anti-inflammatory and immune-modulatory agent commonly used to treat inflammatory arthritis and bowel disease systematically (Rains et al., 1995; Gan et al., 2005). SSZ has been found to inhibit VSMC proliferation and reduce NH (Kim et al., 2009). However, the underlying mechanisms of action of SSZ and autophagy during vascular remodeling after injury have not been fully defined, and their possible therapeutic effects on post-injury vascular restenosis remain to be determined.

The objective of this study is to investigate the potential of SSZ in preventing restenosis. Specifically, we aim to determine whether SSZ can inhibit VSMC growth and migration *in vitro* and *in vivo* following artery injury. Moreover, we aim to elucidate the potential mechanism of SSZ in inducing autophagy.

## Methods

### Synthesis and characterization of sulfasalazine -containing nanoparticles

Poly (lactic-co-glycolic acid) nanoparticles were synthesized through an established emulsion method and were used to encapsulate SSZ (Almería et al., 2011; Park et al., 2011; Steenblock et al., 2011). To elaborate, 100 mg of Poly (lactic-co-glycolic acid) with an inherent viscosity of 0.55–0.75 dL/g (ester terminal) and 10 mg of SSZ were dissolved in chloroform and slowly added drop-wise to a 5% solution of poly (vinyl alcohol) (Sigma-Aldrich). The mixture was sonicated three times and subsequently added to a 0.2% poly (vinyl alcohol) solution. Under stirring, the solvent was evaporated for 2 h, and the poly (lactic-co-glycolic acid) particles were centrifuged prior to lyophilization. The concentration of SSZ was determined by spectrophotometry (SpectraMax M5, Molecular Devices) at 565 nm. Pluronic F-127 (Sigma-Aldrich) was dissolved in PBS over a duration of 3 days at 4 °C, and the nanoparticles were added (50 mg particles/mL, 50 μg SSZ/mg NP [50% loading efficiency], mean NP diameter 380.5 nm; mean zeta-potential −38.8 ± 2.5 mV).

### Kinetic analysis of sulfasalazine-containing nanoparticles

Sulfasalazine -containing nanoparticles (NP-SSZ) in pluronic gel were placed in a microdialysis system and the release of SSZ was monitored by periodic withdrawal of PBS samples. This was also performed with NP-SSZ without pluronic gel. The release was studied by placing a dispersion of NP-SSZ into a dialysis bag (cut-off 20,000 g/mol, Millipore) and immersing the bag into a vial containing PBS (1% Tween, 10 mL). At appropriate times, 1 ml of the release medium was collected from the vials and the medium was replaced by fresh PBS.

### Animals and models

All animal laboratory studies were conducted in compliance with the guidelines on animal care of the Second Xiangya Hospital of Central South University, and were approved by the Institutional Animal Care and Use Committee. Male mice (C57BL/6J) aged 8–10 weeks and weighing between 23 and 30 g were used for this experiment. The mice were housed in a room maintained at 22 °C with a 12-h light/dark cycle, and provided with *ad libitum* access to food and water. The carotid artery procedure was performed as described in previous studies (Williams et al., 2022). Briefly, the mice were anesthetized with an intraperitoneal injection of 100 mg/kg ketamine-HCl and 10 mg/kg xylazine-HCl. Both the left and right carotid arteries were exposed, and the left common carotid artery was ligated proximal to the bifurcation, while the right carotid artery was subjected to a sham procedure. For the NP-SSZ treatment group, NP-SSZ were dissolved in pluronic gel and applied to the ligated carotid artery adventitia prior to closing the skin. For the control group, only equivalent amount of NPs were dissolved in pluronic gel and applied to the ligated carotid artery adventitia prior to closing the skin.

### Histology

For histological analysis, the carotid arteries were excised and embedded in paraffin and 5 μm-thick transverse sections on the glass slides were stained with hematoxylin and eosin (H&E), Elastica van Gieson (EVG), immunofluorescence was performed, according to the standard protocols; six sections taken from the middle portion of each artery, 28 days after the carotid artery injury, were examined; The histological sections of the neointimal area, medial area, and neointima/media (NI/M) ratio were analyzed using ImageJ software. Anti-Ki67, α-SMA, SM22α, MMP2, MMP9, Collage III, IBA-1 antibodies (all from Abcam), CD31/PECAM-1 (R&D), and other fluorescent secondary antibodies were obtained from Invitrogen.

### Western blotting

Protein was extracted from carotid arteries and SMCs using RIPA lysis buffer containing a protease inhibitor cocktail (Thermo Scientific) with PhosSTOP (Roche) and boiled in SDS sample buffer for 6 min. Equal amounts of protein per sample were separated by SDS-PAGE, transferred electrophoretically to a nitrocellulose membrane (Bio-Rad Laboratories), and blotted with primary antibodies, followed by horseradish peroxidase-conjugated secondary antibodies (Cell Signaling). Primary antibodies: anti‐p‐mTOR, anti‐mTOR, anti‐LC3B, anti‐P62 all from Abcam, HSP90 (Santa Cruz), Phospho-NF-κB p65 (Ser536), NF-κB p65, GAPDH, secondary antibody: anti-mouse HRP, anti-rabbit HRP all from Cell Signaling.

### Quantitative real-time polymerase chain reaction assay (qRT-PCR)

Crushed carotid arteries or scraped cells were immersed in lysis buffer and total RNA was extracted using TRIzol (Invitrogen) according to the manufacturer’s instructions. RevertAid First-Strand cDNA Synthesis Kit (Thermo, Waltham, MA, United States of America) was performed for reverse transcription according to the manufacturer’s specifications. Subsequently, SYBR Green-based real-time PCR was performed in triplicate using SYBR Green master mix (Thermo Fisher Scientific) on an Applied Biosystems StepOnePlus real-time PCR machine (Thermo Fisher Scientific). For analysis, the threshold cycle (Ct) values for each gene were normalized to expression levels of GAPDH. Analysis was performed using the Bio-Rad CFX Manager software. Primer sequences were mouse GAPDH: forward, 5′-TGA​AGC​AGG​CAT​CTG​AGG​G-3′, reverse 5′-CGA​AGG​TGG​AAG​AGT​GGG​AG; TNF-α: forward, 5′-CCT​CCC​TCT​CAT​CAG​TTC​TA-3′, reverse,5′-ACTTGGTGGTTTGCTACGAC-3′ MCP-1: forward, 5′-CTT​CTG​GGC​CTG​CTG​TTC​A-3′, reverse, 5′-CCA​GCC​TAC​TCA​TTG​GGA​TCA-3′, ICAM-1: 5′-CAA​TTT​CTC​ATG​CCG​CAC​AG-3′, reverse, 5′-AGC​TGG​AAG​ATC​GAA​AGT​CCG-3′; VCAM-1: forward, 5′-CTT​CAT​CCC​CAC​CAT​TGA​AG-3′, reverse, 5′-TGA​GCA​GGT​CAG​GTT​CAC​AG-3′.

## Cell migration assay

### Wound healing

HA-VSMC cells were seeded in six-well plates at 2.5 × 10^5^ cells/well and cultured until cell monolayers formed. Monolayers were wounded in the center of the confluent cell monolayer by manual scraping with a 200-μL micropipette tip and washed with fresh media. The cells were then incubated with fresh medium supplemented with or without SSZ (200uM SSZ) for 24 h. Wound repair was analyzed by measuring the injured area covered by cells counted from the edge of the scratched lesion with the ImageJ software.

### CCK‐8 assay

CCK‐8 Assay Kit (MedChem- Express) was used for assessing the viability of the VSMCs. The cells were seeded in the 96‐well plate, followed by different treatments. Then the cells were incubated with CCK‐8 reagent at 37 °C in the dark according to the manufacturer’s instructions. The absorbance was measured at 450 nm using a microplate reader.

### Statistics

Data are represented as mean value ±SEM. All data were analyzed using Prism 8 software (GraphPad Software, Inc., La Jolla, CA). Data were assumed to be from a normal distribution with equal variance, as suggested by normality (D’Agostino and Pearson test or Shapiro-Wilk test) and F tests. Statistical significance for these analyses was determined using Student t test or ANOVA (with repeated measures for time series data) with the Sidak *post hoc* correction. *p* values < 0.05 were considered statistically significant.

## Results

### Controlled release of sulfasalazine-containing nanoparticles

**FIGURE 1:**
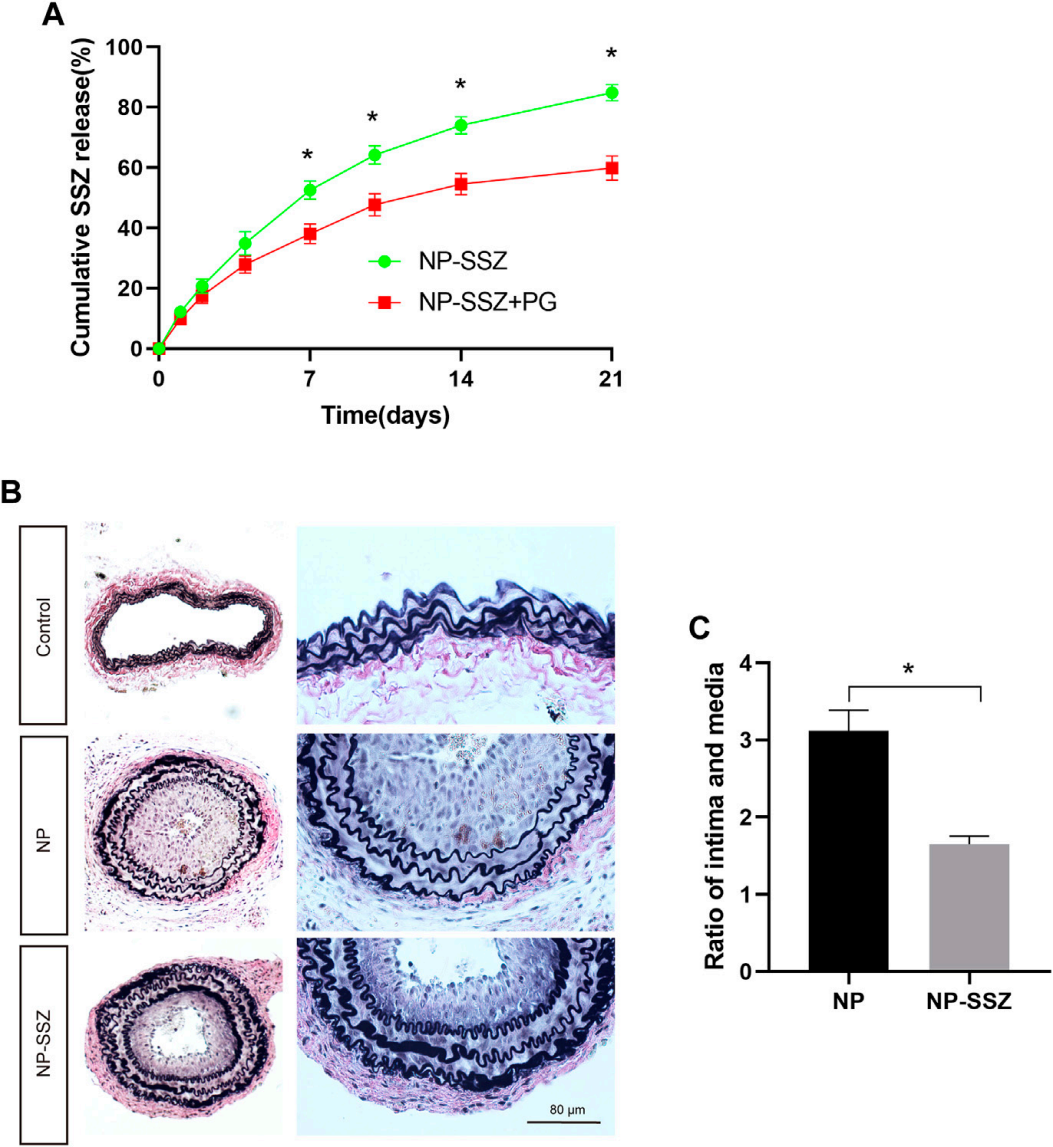
NP-SSZ inhibited injury-induced intimal hyperplasia. **(A)** Controlled release of SSZ-containing nanoparticles *in vitro*; Elution curve showing SSZ release from NP-SSZ (green) or NP-SSZ in pluronic gel (red) over 21 days *in vitro*; *p* < 0.0001 (ANOVA); *p < 0.0001 (days 7–21, Sidak’s *post hoc*); n = 6 each. **(B)** Representative images of EVG staining of carotid arteries in different groups. **(C)** Quantitative analysis of EVG staining of carotid arteries in different groups. EVG: Elastin van Gieson stain. Scale bar: 80 μm *p < 0.05 *versus* the control group. All data are represented as mean value ± SEM.

The pharmacokinetics of SSZ release from NP-SSZ was assessed *in vitro*; cumulative release of SSZ was sustained for at least 21 days (Figure 1A; green). In addition, NP-SSZ that were dissolved in pluronic gel showed less cumulative release of SSZ, with approximately 55% by day 21 (Figure 1A; red). These data show that NP-SSZ that were dissolved in pluronic gel have a longer release time could be better with a single dose delivered to the adventitia at the time of surgery.

### NP-SSZ attenuates neointima hyperplasia and ECM deposition

**FIGURE 2:**
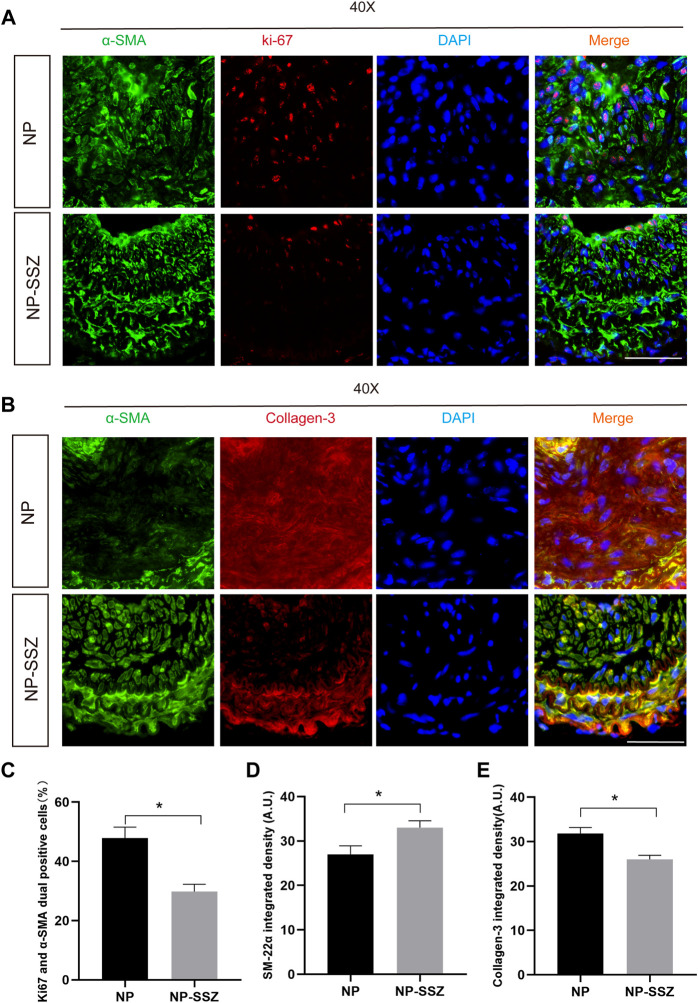
NP-SSZ inhibited injury-induced VSMCs proliferation and promotes synthetic VSMC characteristics. **(A)** Representative images of immunofluorescence staining of Ki67 in the carotid arteries. **(B)** Representative images of immunofluorescence staining of SM22α and Collagen III in the carotid arteries. **(C)** The percentage of Ki67 and α-SMA duel positive cells in the carotid arteries. **(D,E)** Quantitative analysis of SM22α and Collagen III integrated density in different groups. Scale bar: 80 μm *p < 0.05 *versus* the control group.

The injured artery was followed by vascular remodeling which presented prominent luminal narrowing with NH. SMCs play a critical role in NH, including proliferation, phenotypic switching, and ECM deposition. In this study, the NH was measured as the ratio of intima to media (I/M), the ratio of I/M was increased after ligation injury at day 28, while the ratio was significantly reduced in the NP-SSZ treatment group (Figures 1B,C), indicating that NP-SSZ inhibited NH which induced by ligation injury. SMCs proliferation makes a big contribution to the NH, Ki-67, a maker of proliferation, was detected and compared in this study to see if there are any differences between the NP-SSZ treatment group and the control group. The dual positive nuclei of Ki-67 and α-SMA were 47.83 ± 9.15%, whereas only 29.83 ± 5.98% in the SSZ-treated group (*p* < 0.05) (Figure 2 A, C). The immunostaining shows that Ki-67 in SMCs was decreased in the NP-SSZ treatment group, indicating that NP-SSZ inhibited SMCs proliferation.

**FIGURE 3:**
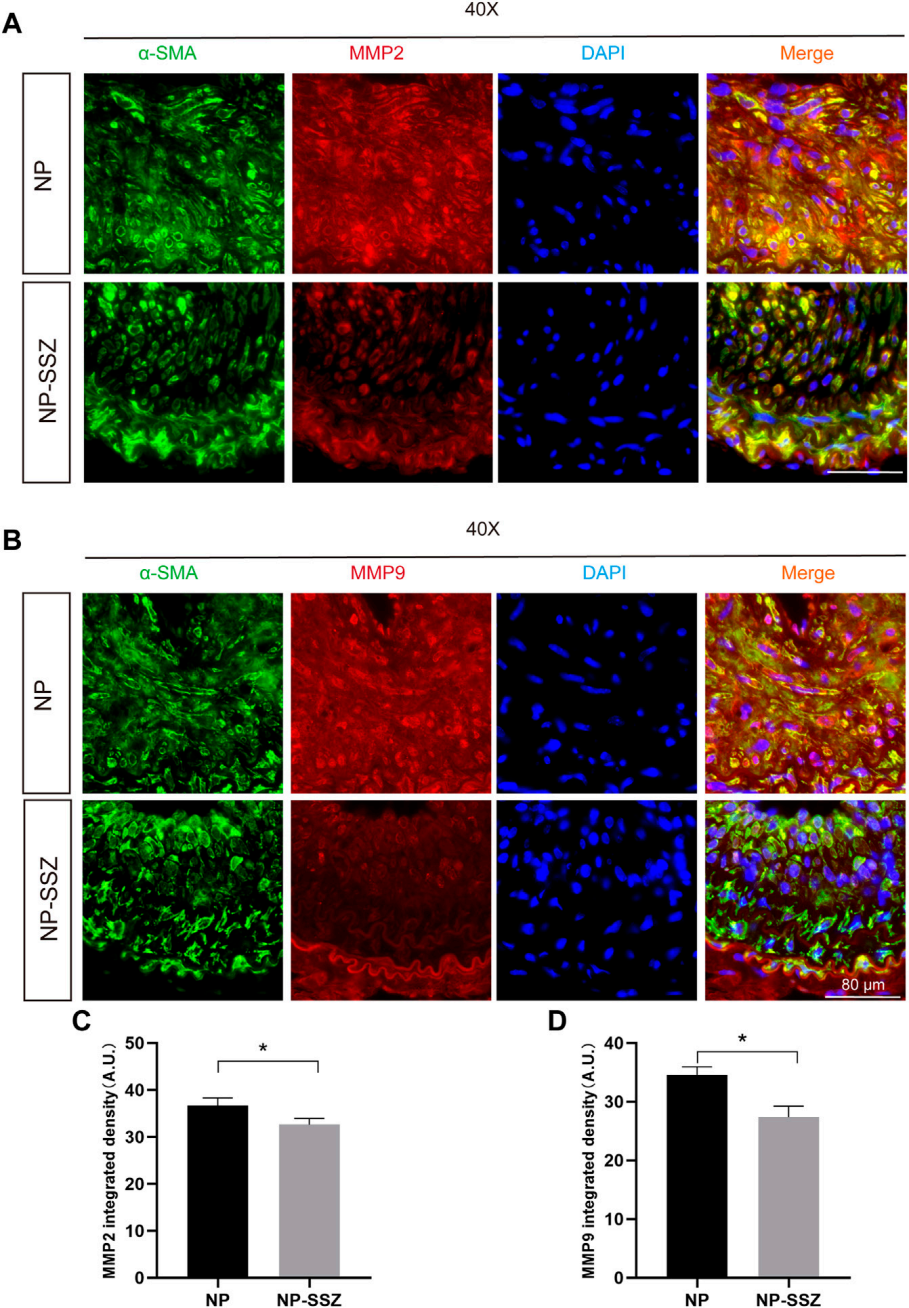
NP-SSZ attenuates ECM deposition. **(A,B)** Representative images of immunofluorescence staining of MMP2 and MMP9 in the carotid arteries. **(C,D)** Quantitative analysis of MMP2 and MMP9 integrated density in different groups. Scale bar: 80 μm *p < 0.05 *versus* the control group.

Since MMP-2 and MMP-9 were abundantly expressed in proliferative neointima, we can detect them as another marker to evaluate the severity of NH in the NP-SSZ treatment group and control group. The immunostaining results show that both the MMP-2 and MMP-9 were decreased in the NP-SSZ treatment group (*p* < 0.05, *p* < 0.05, respectively) compare to the control group (Figure 3). Next, we studied the VSMC characteristics of the injured artery under the NP-SSZ treatment since the MMP family promotes synthetic VSMC characteristics (Newby, 2006). The immunostaining results show that the expression of SM-22α was significantly increased in the NP-SSZ treatment group, while the expression of collagen type III was decreased (Figure 2 B, D, E).

### NP-SSZ suppressed inflammation in response to injury

**FIGURE 4:**
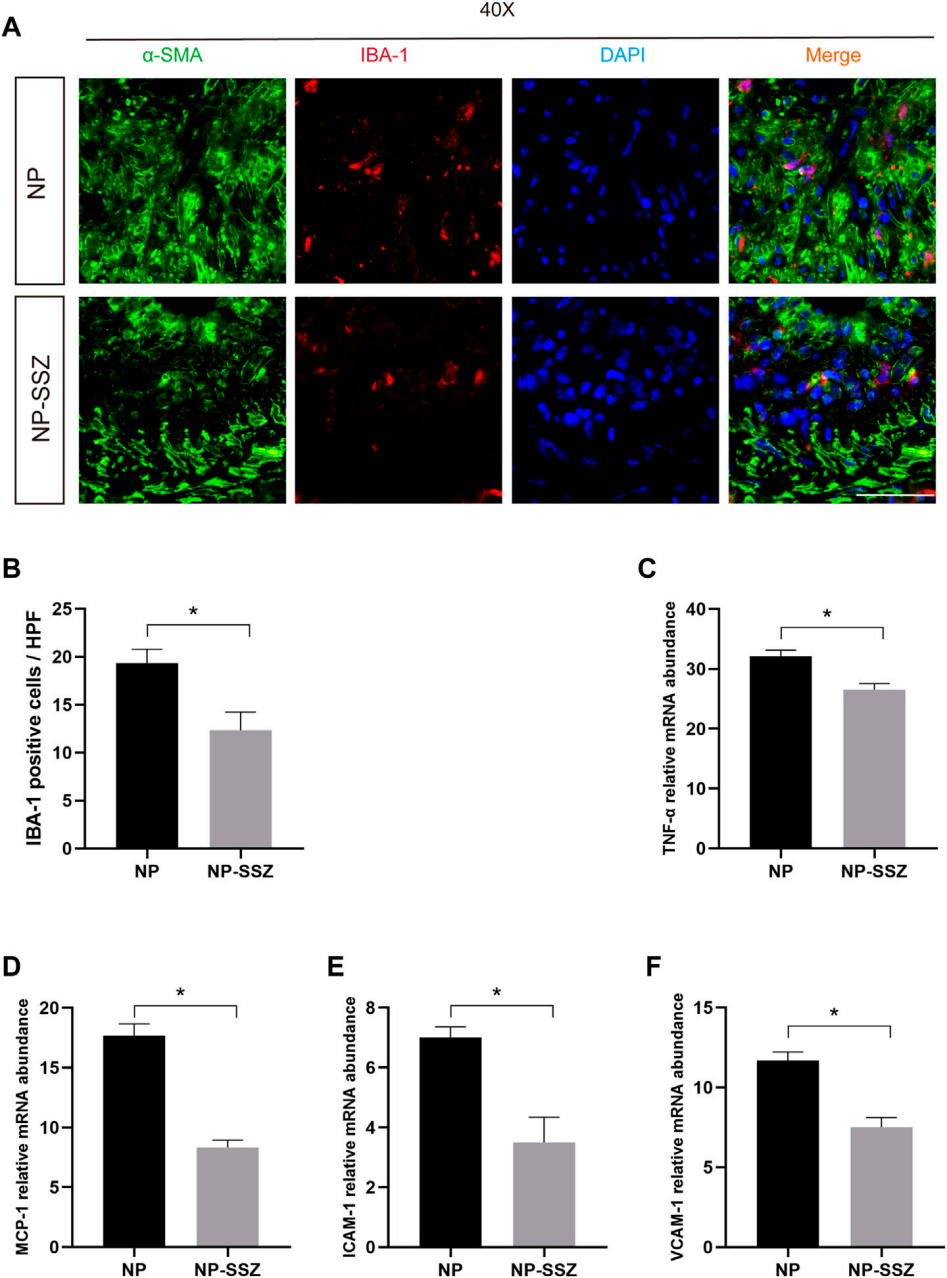
NP-SSZ suppressed inflammation in response to injury. **(A)** Representative images of immunofluorescence staining of IBA-1 in the carotid arteries. **(B)** Quantitative analysis of IBA-1 in different groups. **(C–F)** Quantitative PCR analysis of inflammatory genes of TNF-α, VCAM-1, ICAM-1, MCP-1 in different groups. Scale bar: 80 μm *p < 0.05 *versus* the control group.

The quantitative PCR was performed to investigate whether NP-SSZ can affect the inflammatory response, the results showed that the levels of the targeted inflammatory genes (TNF-α, VCAM-1, ICAM-1, MCP-1) were lower in the NP-SSZ treatment group compared with the control group (Figure 4). Moreover, the IF results showed that the Ionized calcium-binding adaptor molecule 1(IBA-1) was decreased in the NP-SSZ treatment group (*p* < 0.05). Therefore, those data demonstrate that NP-SSZ suppressed the inflammatory response after artery injury.

### SSZ inhibited TNF-α‐induced proliferation and migration of VSMCs *in vitro*

**FIGURE 5:**
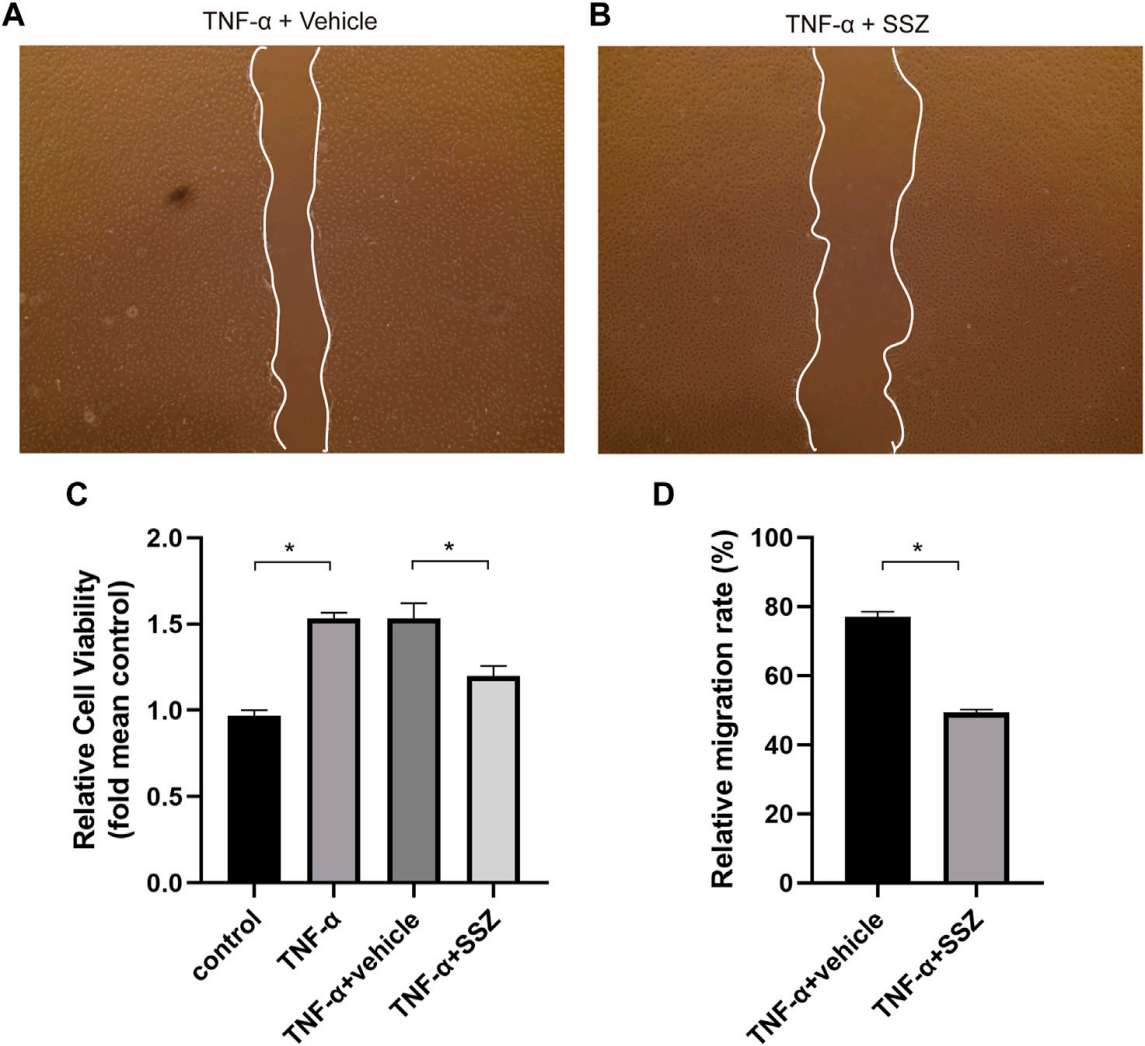
SSZ inhibited TNF-α‐induced migration of VSMCs *in vitro*. **(A,B)** Representative images of migrated cells at 24 h after scratch in the different groups. **(C)** Quantitative analysis of CCK-8 assay. **(D)** Quantitative analysis of cell scratch assay. VSMC, vascular smooth muscle cell. *p < 0.05 *versus* the control group.

**FIGURE 6:**
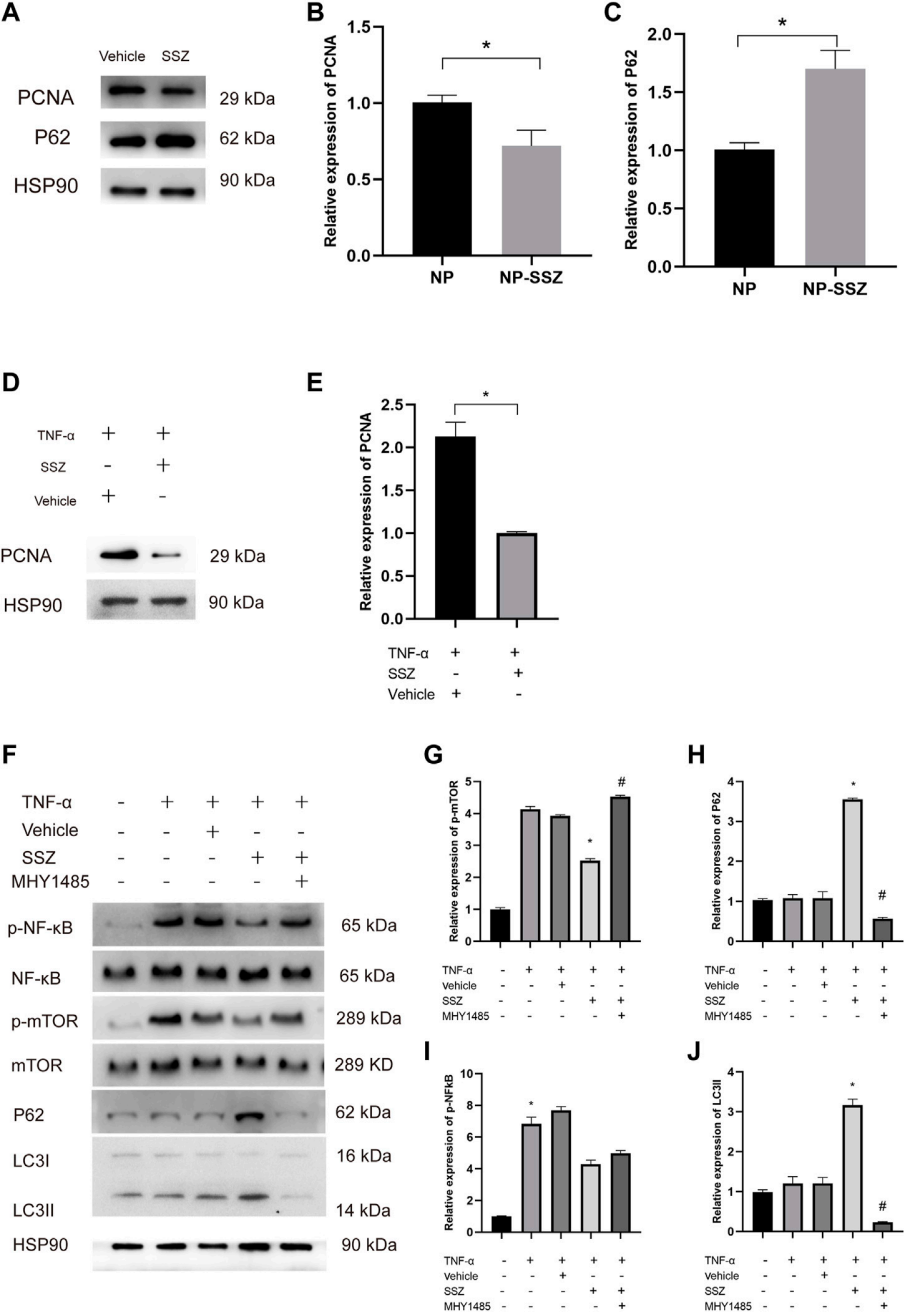
SSZ promotes autophagy via NF-kB/mTOR pathway. **(A)** the protein expression levels of PCNA, and P62 in injured carotid arteries with different treatment were detected by Western blot. **(B,C)** Quantitative analysis of PCNA and P62 proteins expression level. **(D)** the protein expression levels of PCNA in VSMC with or without TNF-α treatment. **(E)** Quantitative analysis of PCNA proteins expression level. **(F)** the protein expression levels of p‐NF-Kb, NF-kB and p‐mTOR, mTOR, LC3 II, and P62 in VSMC with different treatment were detected by Western blot. **(G–J)** Quantitative analysis of p‐NF-kB/NF-kB and p‐mTOR/mTOR as well as LC3 II and P62 proteins expression level. VSMC, vascular smooth muscle cell. *p < 0.05 *versus* the control group.

The proliferation and migration of VSMCs make a great contribution to neointima hyperplasia. VSMCs were treated with TNF-α to induce cell proliferation and migration for investigating the effects of SSZ on VSMCs. The CCK‐8 assay results demonstrated that the cell viability of VSMCs was markedly increased in the TNF-α treatment group (Figure 5C), whereas SSZ treatment inhibited this effect. In addition, a cell scratch assay was performed in the groups of TNF-α+Vehicle and TNF-α+ SSZ group for evaluating the effect of SSZ on VSMCs migration. As reflected in the scratch assay, the migration rate of the TNF-α group was significantly higher than that of the control group, however, the migration rate was decreased in the SSZ treatment group, indicating that SSZ suppressed migration of VSMCs (Figure 5D). Western blot was performed to evaluate the proliferation of VSMCs. Compare to the vehicle group, the PCNA expression was significantly decreased in the SSZ treatment group, which was consistent with the results *in vivo* (Figure 6A). The above results suggested that the SSZ treatment can inhibit VSMC proliferation and migration which was induced by TNF-α.

### SSZ promotes autophagy *in vivo* and *in vitro*

It is reported that autophagy inhibits the proliferation and migration of VSMCs to reduce neointima hyperplasia. To investigate whether NP-SSZ induces autophagy in the injured carotid arteries, a Western blot was performed to detect the hallmarks of autophagy. The results showed that P62 protein were higher in the NP-SSZ group than the vehicle group, indicating that NP-SSZ promotes autophagy in carotid arteries after injury (Figure 6A). We use the TNF-α to induce the VSMC proliferation and migration, similarly, the Western blot results showed the LC3 II and P62 protein expression were higher in the group co-treatment with SSZ (Figures 6F,H,J). Taken together, those results demonstrated that SSZ promotes autophagy both *in vivo* and *in vitro*.

### SSZ inhibits NF-kB activation

It is reported that SSZ can inhibit NF-kB activation *in vivo* (Wahl et al., 1998; Robe et al., 2004). In this study, WB was performed to detect the effects of SSZ on NF-kB activation. The WB results showed that P62 expression in the SSZ treatment group was lower than the control group with a significant difference (*p* < 0.05) (Figures 6F,H). Those results demonstrate SSZ inhibits NF-kB activation.

### SSZ promotes autophagy via NF-kB/mTOR pathway

Since SSZ promotes autophagy and previous studies have shown that mTOR plays a pivotal role in autophagy, we hypothesize that SSZ affects the mTOR expression inducing autophagy to inhibit the VSMCs proliferation and migration. To prove this hypothesis, MHY1485, an agonist of mTOR was used to attest whether NF-kB/mTOR pathway is essential for SSZ -inducing autophagy. The Western blot results have shown that the phosphorylation of NF-kB (p‐NF-kB) and the phosphorylation of mTOR (p‐mTOR) were decreased in the TNF-α + SSZ group, whereas the P62 and LC3 II expression levels were increased. However, the expression level of p‐mTOR, P62, and LC3 II was reversed after co‐treatment with MHY1485, whereas the p-NF-kB expression level was without any changes (Figure 6F). Taken together, these data indicate that SSZ promotes autophagy through NF-kB/mTOR pathway.

## Discussion

Arterial injury-induced neointimal hyperplasia (NH) is a major cause of stenosis. Vascular smooth muscle cell (VSMC) proliferation, migration, and extracellular matrix (ECM) deposition are key contributors to NH (Cheng et al., 2002). Thus, identifying effective therapeutic strategies for preventing VSMC proliferation and neointimal formation is critical for treating vascular stenosis after arterial injury.

Previous research has demonstrated that matrix metalloproteinases (MMPs) play a significant role in promoting NH by enhancing the synthetic properties of VSMCs and migration (Newby, 2006; Wang et al., 2015). In the current study, we observed a decrease in MMP-2 and MMP-9 expression in the NP-SSZ treatment group, indicating that NP-SSZ inhibits MMP expression and reduces VSMC synthetic properties, ultimately suppressing NH. Additionally, we observed an increase in the VSMC contractile marker SM-22α and a decrease in the synthetic marker Collagen III following NP-SSZ treatment.

NH is a complex, multifactorial pathological process involving VSMC activation, inflammation, and oxidative stress (Bonta et al., 2010; Pi et al., 2013). Although the expression of MMP-2 and MMP-9 was inhibited by NP-SSZ, these MMPs are mainly expressed and secreted by inflammatory cells (Cheng et al., 2011). Therefore, we investigated whether NP-SSZ can suppress inflammation during vascular remodeling after arterial injury. Our results show that NP-SSZ inhibits the expression of inflammatory genes (TNF-α, VCAM-1, ICAM-1, MCP-1) and macrophages, indicating that NP-SSZ suppresses inflammation during vascular remodeling. Thus, in addition to inhibiting VSMC proliferation and migration, NP-SSZ also ameliorates the inflammation response.

Autophagy is a multifactorial and pathological cellular process that removes dysfunctional components and is considered a non-selective degradation mechanism (Kim et al., 2008). Autophagy plays a crucial role in NH (Luo et al., 2018), and previous studies have found that autophagy has a negative correlation with VSMC proliferation and migration (Wang et al., 2019). Therefore, we examined the autophagy markers in the NP-SSZ treatment group and control group to see if NP-SSZ has an effect on autophagy. Consistent with previous studies, our results indicate that NP-SSZ enhances autophagy and inhibits VSMC proliferation and migration.

SSZ specifically inhibits the activation of NF-kB, resulting in the downregulation of inflammatory cytokine mRNA expression (Rains et al., 1995; Gan et al., 2005). Additionally, SSZ has been shown to inhibit NF-kB activation, block the cell cycle, and induce apoptosis in several cancer cell lines (Robe et al., 2004). However, the underlying mechanisms by which SSZ promotes autophagy effects remain unclear. In this study, we found that SSZ enhances VSMC autophagy while inhibiting NF-kB and mTOR activation. Moreover, mTOR is a key protein regulating autophagy and VSMC proliferation and migration (Daskalopoulos et al., 2016; Chen et al., 2020). To understand the mechanism of SSZ promoting autophagy involving the NF-kB and mTOR, we used an agonist of mTOR and found that it promoted mTOR activation and inhibited autophagy without affecting NF-kB activation. Therefore, the major mechanism of action of SSZ in regulating autophagy may be through NF-kB/mTOR inhibition.

In conclusion, this study provides evidence that SSZ enhances autophagy and suppresses NH by inhibiting VSMC proliferation and migration through the NF-kB/mTOR pathway.

## Data Availability

The original contributions presented in the study are included in the article/Supplementary Material, further inquiries can be directed to the corresponding author.
