# Supplementary figures and images for: Sulfasalazine induces autophagy inhibiting neointimal hyperplasia following carotid artery injuries in mice
Source: Front Bioeng Biotechnol. 2023 May 23;11:1199785. doi: 10.3389/fbioe.2023.1199785 (PMC10242098; doi:10.3389/fbioe.2023.1199785)

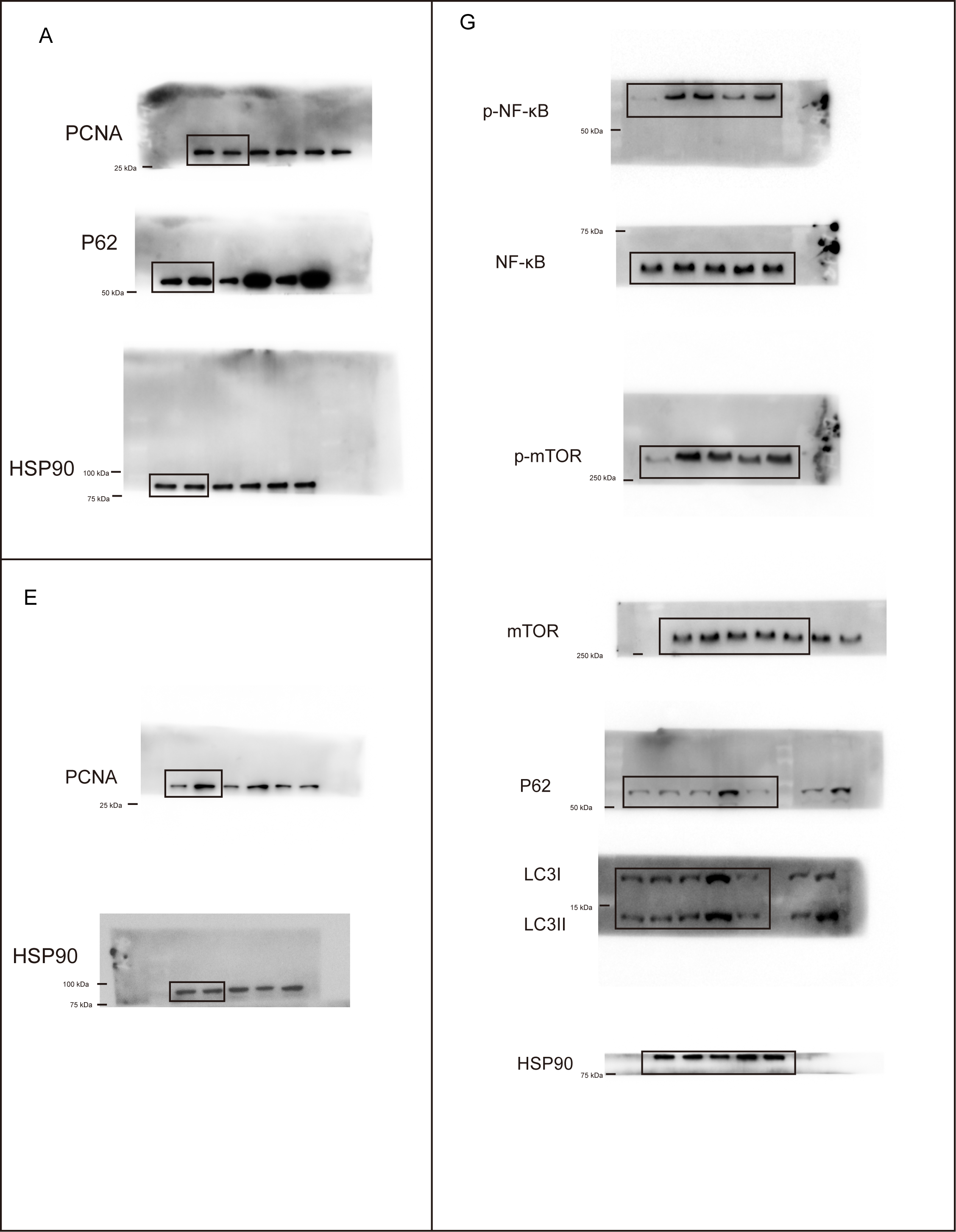

Supplement: Supplementary file 1 [file Image1.TIF]
